# Emerging Trends in Green Extraction Techniques, Chemical Modifications, and Drug Delivery Systems for Resveratrol

**DOI:** 10.3390/antiox14060654

**Published:** 2025-05-29

**Authors:** Sonia Trombino, Roberta Cassano, Maria Luisa Di Gioia, Francesca Aiello

**Affiliations:** Department of Pharmacy, Health and Nutritional Science, University of Calabria, Arcavacata, 87036 Rende, Italy; sonia.trombino@unical.it (S.T.); roberta.cassano@unical.it (R.C.)

**Keywords:** resveratrol, green extraction, green solvents, structure-activity relationships, resveratrol derivatives, drug delivery systems

## Abstract

Resveratrol is a naturally occurring phytoalexin found in red grapes, cocoa berries, and red grape wine. This compound exhibits potent antioxidant, anti-inflammatory, and anticancer properties. However, its clinical application is significantly hindered by poor aqueous solubility and rapid degradation at physiological pH, resulting in extremely low systemic bioavailability. This review explores three key aspects: green extraction methods for the efficient and sustainable isolation of resveratrol; structure–activity relationship studies of resveratrol derivatives to identify compounds with improved bioavailability and therapeutic efficacy; and advanced drug delivery systems to enhance resveratrol solubility, stability, and achieve targeted tissue delivery. All of these solutions collectively aim to increase resveratrol bioavailability, enabling the development of effective pharmaceutical formulations and maximizing the clinical potential of this promising compound. The aim of this review is to summarize the key studies published in the last five years, highlighting innovative advancements in sustainable extraction, structural modifications, and delivery strategies for resveratrol.

## 1. Introduction

**Figure 1:**
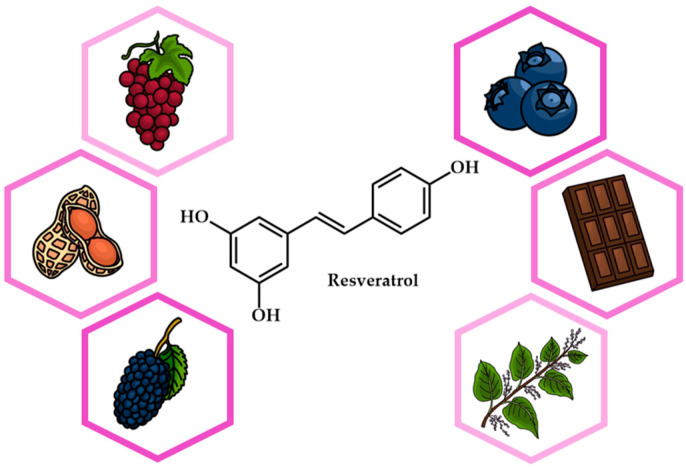
Chemical structure and natural sources of resveratrol.

Resveratrol (RSV) first came to prominence when it was extracted from *Veratrum grandiflorum*, commonly known as the white hellebore plant. In plants, RSV is synthesized via the phenylpropanoid pathway, starting with the amino acid phenylalanine. Naturally occurring in foods such as red grapes, berries, peanuts, and dark chocolate (Figure 1), RSV has multiple effects on apoptosis, oxidative stress, and inflammation. It targets numerous pathways, including NF-kB and PI3K/Akt, signaling its potential as a promising drug candidate for conditions such as cardiovascular disease, diabetes, and cancer [1,2,3]. Clinical trials have investigated the safety of RSV, suggesting that doses of up to 5 g per day are generally well tolerated. However, its low bioavailability due to its quick metabolism in the intestine and liver can limit its clinical efficacy. In addition, the variability in bioavailability between individuals is influenced by factors such as age and gender. When used topically, RSV is considered potentially safe for up to 30 days. Similarly, when used as a nasal spray, it is potentially safe for up to four weeks. In individuals with cardiovascular disease and heart failure, doses of RSV vary from 8 mg/day to 1500 mg/day. For cancer prevention, recommended daily doses of RSV range from 0.5 to 1.0 g, with the aim of exploiting its potential anticancer properties.

Due to its widespread availability and biological significance, resveratrol has been the focus of extensive research over the past decade. Numerous studies have explored various extraction techniques, ranging from conventional to advanced technological approaches, with the objective of maximizing polyphenol recovery. Conventional extraction techniques present several drawbacks, including low extraction efficiency, extended processing times, and environmental concerns related to solvent toxicity and waste generation [4]. To address these challenges, the development of sustainable, green extraction technologies has become a priority. The integration of environmentally friendly extraction techniques represents a crucial step toward the sustainable production of bioactive compounds, aligning with the principles of the circular economy and sustainable development.

Despite the promising therapeutic potential of RSV, it is important to note that the data are insufficient to fully establish the efficacy and risks of resveratrol supplements. Side effects are generally rare, but some people may experience intestinal upset and nausea. Additionally, high doses of RSV may inhibit enzymatic activity, which may increase the bioavailability and toxicity of some drugs. So far, the best way to obtain safe amounts of RSV has been found to be through dietary sources, such as grapes, wine, blueberries, cranberries, pomegranates, and juices containing these fruits [5].

Given the impressive therapeutic potential of RSV, significant efforts have been dedicated to overcoming its low bioavailability through the synthesis of RSV analogs with improved behavioral and RSV kinetics.

Furthermore, the incorporation of resveratrol into controlled drug delivery systems represents a valid strategy to address its limitations. These systems allow for the controlled release of RSV at a predefined rate, maintaining prolonged therapeutic concentrations or facilitating targeted release to the site of action. This approach minimizes adverse effects and optimizes bioavailability while maximizing the therapeutic efficacy of RSV. These strategies aim to develop effective pharmaceutical formulations that expand the clinical potential of this promising compound [6,7,8,9]. 

This review provides an overview of recent eco-friendly strategies for resveratrol (RSV) recovery, with a particular emphasis on green extraction techniques, chemical modifications, and pharmaceutical applications. Given the large number of reviews already published on the general properties and health benefits of resveratrol [10,11,12,13,14,15,16], the scope of this work has been intentionally narrowed to these three specific aspects, thereby enabling a more detailed and up-to-date analysis of recent technological and scientific advancements.

## 2. Information Sources

A literature search was performed, inspecting the most relevant scientific databases, i.e., Scopus and Web of Science (WOS), over the last five years. The systematic literature search, the screening of the retrieved records, and the selection of results were derived from the use of the following key phrases: importance of resveratrol; health benefits of resveratrol; potential therapeutic applications: mechanism of action at the molecular level; bioavailability and safety; natural sources of resveratrol; dietary sources of resveratrol; environmentally friendly extraction methods for resveratrol; advantages of using green solvents in resveratrol extraction; challenges and limitations of using green solvents in resveratrol extraction; nanoparticles drug delivery systems; enhancement of bioavailability; challenges in developing resveratrol drug delivery systems; resveratrol derivatives: synthesis and their biological activities.

## 3. Environmentally Friendly Extraction Methods for Resveratrol

RSV is an aromatic polyphenol naturally produced by various plant species. It has been identified in over 100 plants across 34 botanical families, including *Vitaceae*, *Moraceae*, and *Leguminosae* [17,18]. Structurally, RSV exists in two isomeric forms, namely, *cis*-resveratrol and *trans*-resveratrol, which can interconvert under the influence of environmental factors such as light, temperature, and pH. The *trans*-isomer is the more stable and bioactive form, exhibiting strong antioxidant properties. As a phytoalexin, RSV plays a crucial role in plant defense, being synthesized in response to stress conditions such as microbial infections or environmental stressors [19]. Its production is often upregulated under adverse conditions to enhance the plant’s resilience. Currently, red grapes are the primary commercial source for RSV extraction [10]. The concentration of RSV in fresh grape skins is estimated to range from 5 to 10 × 10^−2^ g/kg. In red wine, RSV levels typically vary between 1.5 and 3 mg/L, though some studies report higher concentrations ranging from 4 to 20 mg/L, with notable levels also detected in certain white and rosé wines.

**Figure 2:**
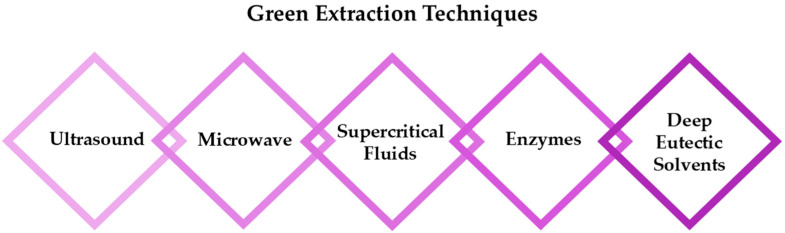
The most promising approaches for successful extraction of RSV.

The selection of an appropriate extraction technique is crucial as it directly influences the chemical stability, functionality, and potential toxicity of RSV, in addition to environmental considerations such as energy consumption, heat generation, and waste production [20]. While traditional extraction methods, such as liquid extraction, percolation, and organic solvent extraction, continue to be widely employed, considerable advancements have been made to improve these techniques through modern technologies (Figure 2) [11,21,22]. Innovations like ultrasound-assisted extraction (UAE), microwave-assisted extraction (MAE), and supercritical fluid extraction (SFE) have demonstrated superior efficiency, reduced solvent consumption, and better preservation of bioactive compounds. Additionally, enzymatic extraction has gained attention as a promising alternative due to its high selectivity, increased recovery rates, and gentle processing conditions. Recently, the use of deep eutectic solvents (DES) [23,24] as eco-friendly substitutes for traditional solvents has emerged as a promising approach, further enhancing the efficiency and sustainability of resveratrol (RSV) recovery.

Table 1 provides an overview of recent environmentally friendly methods for RSV extraction, highlighting advancements in sustainable technologies.

Among the most widely applied emerging extraction techniques, MAE and UAE have attracted attention due to their efficiency and sustainability [25,26]. MAE offers distinct advantages due to its ability to provide rapid and uniform heating while minimizing undesirable secondary reactions compared to conventional extraction methods. This technique utilizes non-ionizing electromagnetic waves to induce dipolar rotation and ionic conduction, generating heat from within the material itself. As a result, MAE enhances both mass and heat transfer, leading to accelerated extraction rates [27,28]. Studies have shown that MAE, particularly when combined with water as a solvent, significantly improves polyphenol yields compared to traditional Soxhlet extraction [29].

UAE also presents multiple advantages over conventional methods, including reduced operational costs, shorter extraction times, and lower operating temperatures. Working under milder conditions helps preserve the integrity of sensitive bioactive compounds. UAE operates through acoustic cavitation, a phenomenon that disrupts cell walls and enhances the diffusion of bioactive compounds. This technique is widely implemented at both laboratory and industrial scales, utilizing ultrasonic baths for indirect sonication or ultrasonic probes for direct sonication [30]. A recent study by Munoz-Realpe et al. [31] investigated the extraction of resveratrol from “Charelo” vine shoots. The extraction was optimized using probe UAE, and MAE and was evaluated through response surface methodology. Ultrasound extraction (62% amplitude, 6 min, 59% ethanol, 55 °C) yielded 60 mg/g of extract with 1.05 mg/g trans-resveratrol. Microwave extraction (80 °C, 4 min, 69% ethanol) provided a slightly smaller extract but with higher phenolic content, including 1.32 mg/g *trans*-resveratrol. At 155 °C, extract yield and phenolic content increased but with higher energy consumption. Additionally, Zhou et al. [32] used ultrasound-assisted aqueous two-phase extraction (UAATPE) to extract and concentrate RSV from enzymatic hydrolysates of *Polygonum cuspidatum.* This technique employed an ethanol–ammonium sulfate system, optimizing the RSV yield to 10.7 mg/g, with a remarkable recovery rate of 99.1%. Compared to conventional UAE and solvent extraction methods, UAATPE enhanced RSV recovery by 43.6% and 79.4%, respectively, while minimizing the co-extraction of sugars. Key advantages of this method include its high efficiency, its superior selectivity, and its ability to operate at moderate temperatures, further reinforcing its potential as a sustainable extraction method.

Jin et al. [33] developed an ultrasound-assisted surfactant aqueous solution system integrated with a cellulose-immobilized microbial community (consisting of Yeast CICC 1912, *Aspergillus oryzae* 3.951, and *Aspergillus niger* 3.3148) for the bioconversion and extraction of RSV from peanut skin. The optimized conditions included a 3% (*w*/*v*) surfactant concentration, a liquid-to-solid ratio of 25:1 mL/g, an ultrasonic power of 250 W, a temperature of 30 °C, and an incubation period of 36 h. Under these conditions, the RSV yield reached 96.58 µg/g, representing a 4.02-fold increase compared to untreated samples. This approach demonstrated high efficiency, environmental sustainability, and cost-effectiveness.

Studies focusing on MAE and UAE techniques underscore their energy efficiency while acknowledging challenges like scalability limitations, the risk of compound degradation, and the requirement for additional purification steps in industrial applications.

Supercritical fluid extraction (SFE) has emerged as an environmentally sustainable and highly efficient technique for isolating bioactive compounds, offering key advantages such as solvent-free processing, high selectivity, and minimal thermal degradation [34]. Due to its non-toxic, non-flammable, and cost-effective nature, CO_2_ is the most commonly used solvent in SFE, with its critical points being at 31.06 °C and 7.386 MPa, allowing it to exhibit both gas-like diffusivity and liquid-like solvating power. This unique property enables SFE-CO_2_ to extract thermolabile compounds with high purity while eliminating the need for additional refining steps. Moreover, the use of polar cosolvents, such as ethanol and water, further enhances the extraction efficiency of phenolic compounds, making SFE-CO_2_ a versatile tool in food, pharmaceutical, and cosmetic industries. Zhabayeva et al. [35] investigated the application of SFE for the isolation of RSV from *Vitis vinifera* L. Their study indicated that SFE-CO_2_ extraction at 20 MPa and 60 °C for 3 h optimally enhances RSV yield, whereas increasing pressure beyond 20 MPa or temperature beyond 65 °C negatively impacts recovery. Similarly, the study by Tuhanioglu et al. [36] optimized SFE-CO_2_ extraction conditions for RSV recovery from muscadine grape pomace using ethanol-water as a cosolvent. The process was refined through response surface methodology, identifying optimal conditions at 20 MPa, 60 °C, and 15% cosolvent concentration. Under these parameters, RSV yield reached 1.07 mg/100 g, with total phenolic and flavonoid content comparable to conventional solvent extractions. While SFE-CO_2_ extraction produced lower resveratrol yields than HCl–methanol extraction, it offered a greener, food-grade alternative with minimal solvent waste.

Enzymatic extraction has emerged as a promising green technology for RSV recovery due to its high selectivity, efficiency, and mild processing conditions. This method involves the use of enzymes such as cellulases, pectinases, and glucosidases, which break down complex plant cell wall structures, thereby facilitating the release of RSV. Averilla et al. [37] demonstrated that a combined thermal and enzymatic treatment significantly improved RSV yield from grape peel. The process involves pre-heating at 95 °C for 10 min, followed by enzymatic hydrolysis with β-glucanase and pectinase at 50 °C for 60 min. This method resulted in a 50% increase in RSV extraction, primarily by converting its glucoside precursor into its more bioavailable aglycone form. Similarly, Ping et al. [38] optimized an enzymatic-homogenate synergistic extraction method for RSV recovery from *Rhodomyrtus tomentosa* fruit. By integrating β-glucosidase with homogenization and a natural surfactant, this approach significantly enhanced RSV extraction efficiency while maintaining eco-friendly conditions.

Emerging green solvents for extracting bioactive compounds from natural resources have attracted the interest of researchers. Ionic liquids (ILs) and deep eutectic solvents (DESs) have been extensively studied for their ability to selectively extract polyphenols, including RSV, from plant matrices. Ionic liquids, composed of organic cations and inorganic or organic anions, offer advantages such as low vapor pressure, high thermal stability, and tunable solvating properties. The study proposed by Zhao et al. [39] evaluated various ionic liquids with different carbon chains and anions, optimizing seven key parameters: pH value, enzyme concentration, extraction temperature, extraction time, ultrasonic power, IL concentration, and liquid–solid ratio. The optimal conditions were determined to be an enzyme concentration of 2.18%, an extraction temperature of 58 °C, a liquid–solid ratio of 29 mL/g, a pH value of 5.5, an extraction time of 30 min, an ultrasonic power of 250 W, and a 0.5 mol/L 1-butyl-3-methylimidazolium bromide as the extraction solvent. Under these conditions, the RSV yield was 2.90 ± 0.15 mg/g.

Dimitrijević et al. [40] investigated the use of cholinium-based ionic liquids combined with polypropylene glycol (PPG400) to create aqueous biphasic systems (IL-ABSs) for the extraction of polyphenols from grape by-products. The researchers found that resveratrol preferentially partitioned into the hydrophobic PPG-rich phase. The optimized extraction conditions demonstrated high efficiency, with cholinium dihydrogen phosphate achieving approximately 99% extraction efficiency for RSV.

Recently, concerns regarding ILs biodegradability and potential toxicity have led to an increasing focus on deep eutectic solvents (DES), a subclass of ILs that are composed of natural, biodegradable, and often food-grade components. DESs, formed by hydrogen bond interactions between hydrogen bond acceptors (HBAs) and hydrogen bond donors (HBDs), exhibit remarkable advantages in terms of sustainability, ease of preparation, and cost-effectiveness.

*Polygonum cuspidatum* is a traditional perennial herb recognized in the Pharmacopoeia of The People’s Republic of China. It is commonly used to treat conditions such as arthralgia, chronic bronchitis, jaundice, hypertension, and hypercholesterolemia. Research has indicated that the dried root of *P. cuspidatum* contains significantly higher levels of resveratrol compared to other plant sources. However, RSV is predominantly present as its glycoside, polydatin, which is found in concentrations 10 to 15 times higher than RSV itself. Consequently, developing efficient methods for extracting and converting polydatin to resveratrol from *P. cuspidatum* is a promising strategy to achieve high yields of resveratrol. Sun et al. [41,42] and Shaohua Li et al. both explored innovative one-pot extraction methods utilizing DES for RSV production by converting polydatin to resveratrol.

Sun et al. used hydrochloric acid hydrolysis of polydatin and DES-based extraction using choline chloride–oxalic acid, achieving an extraction yield of 12.26 ± 0.14 mg/g and a conversion efficiency of over 96.3% for polydatin. In contrast, the study by Shaohua Li et al. combined enzymatic hydrolysis with DES extraction, using choline chloride–urea, optimizing conditions to achieve a RSV yield of 19.53 ± 0.22 mg/g and a conversion rate of 100% for polydatin. Both methods highlight the efficiency and environmental benefits of using DES, but the latter study demonstrated a higher yield and conversion rate, showcasing the potential of enzyme-assisted DES extraction for maximizing RSV production from *Polygonum cuspidatum*.

Recent studies have demonstrated the effectiveness of natural deep eutectic solvents (NADES) for RSV extraction. The study on *Aronia melanocarpa* carried out by Wang et al. [43] explored the use of choline chloride–urea and choline chloride–glycerol as NADES in ultrasound-assisted extraction, significantly enhancing the yield of RSV compared to conventional solvents, while maintaining its biological properties. Meanwhile, research on grape pomace valorization [44] employed pressurized liquid extraction (PLE) with green solvents such as hot water and NADES, specifically choline chloride–oxalic acid and choline chloride–urea. PLE resulted in higher yields, shorter extraction times, and improved automation, demonstrating its feasibility for sustainable phenolic recovery, including thermosensitive compounds. The optimization of polyphenol extraction from grapevine canes [45] identified choline chloride–1,4-butanediol as the most effective NADES, enabling the extraction of high concentrations of stilbenes, particularly *trans*-resveratrol and *trans*-ε-viniferin. Through a Box–Behnken optimization, the optimal conditions—38.2% water content, a solid-to-liquid ratio of 50 mg/500 µL, 80 °C temperature, and 17.2 min of extraction—maximized resveratrol recovery. Among the tested NADES, choline chloride–1,4-butanediol (BCH) and choline chloride–oxalic acid exhibited the highest efficiency in RSV extraction.

A study by Abdel-Monem [46] presents an innovative method based on in-syringe vortex-assisted liquid–liquid microextraction (IS-VA-LLME) using a hydrophobic natural deep eutectic solvent (NADES) composed of α-terpineol and n-octanol (1:1) as the extraction phase. This approach was successfully applied for the simultaneous determination of resveratrol in environmental and biological samples, demonstrating good analytical performance in terms of sensitivity, reproducibility, and recovery. Extraction conditions were optimized using a univariate approach, and the method proved effective even in complex matrices such as human plasma and river water, with the result characterized by a green and sustainable profile. However, some limitations remain, including the need for post-extraction dilution with organic solvents and the use of UV detection, which may offer lower sensitivity compared to mass spectrometry-based techniques. Overall, these studies underscore the advantages of NADES as eco-friendly, high-yield alternatives to conventional solvent-based extractions, supporting the shift toward greener and more sustainable resveratrol recovery processes.

The integration of these green approaches, particularly the combination of deep eutectic solvents (DES) with advanced extraction methods such as ultrasound-assisted extraction (UAE), microwave-assisted extraction (MAE), and enzymatic hydrolysis, represents a crucial step toward the environmentally responsible production of bioactive compounds, aligning with the principles of the circular economy and sustainable development.

**Table 1:** Comparative overview of green extraction techniques for resveratrol.

Extraction Method	RSV Yield (mg/g)	Purity/Selectivity	Advantages	Limitations	References
Ultrasound-Assisted Extraction (UAE)	1.05–2.90	High (selective for phenolics)	- Reduced extraction time and solvent use; - Low operating temperature preserves bioactive compounds; - Scalable to industrial applications.	- Risk of compound degradation at high intensities; - Limited penetration in dense matrices.	Munoz-Realpe et al. [31]; Zhou et al. [32]; Jin et al. [33]
Microwave-Assisted Extraction (MAE)	1.32–2.90	High (efficient, minimal degradation)	- Rapid heating improves extraction efficiency; - enhances polyphenol yield; - Energy-efficient.	- Requires careful parameter optimization to prevent thermal degradation; - High initial equipment cost.	Siller-Sánchez et al. [29]; Munoz-Realpe et al. [31]
Supercritical Fluid Extraction (SFE-CO_2_)	1.07	Very high (solvent-free, minimal refining)	- Solvent-free; - high selectivity; - Minimal thermal degradation; - High-purity extracts.	- Requires high-pressure equipment; - Lower RSV yield than some solvent-based methods.	Zhabayeva et al. [35]; Tuhanioglu et al. [36]
Enzymatic Extraction	2.90–19.53	High (aglycone form enrichment)	- High selectivity; - Mild conditions prevent degradation; - Converts polydatin to RSV.	- Longer processing times; - Enzyme costs; - Batch-to-batch variability.	Averilla et al. [37]; Ping et al. [38]
Ionic Liquid (IL) Extraction	2.90 ± 0.15	High (tunable selectivity)	- High efficiency and tunable solvent properties.	- Potential toxicity and biodegradability concerns.	Zhao et al. [39]; Dimitrijević et al. [40]
Deep Eutectic Solvent (DES) Extraction	12.26–19.53	Very high (100% conversion in optimal cases)	- Eco-friendly; - Highly efficient for RSV recovery; - Facilitates polydatin conversion; - Sustainable and biodegradable; - Improved RSV yield compared to conventional solvents.	- Requires optimization of solvent composition; - High viscosity may impact processing; - Limited large-scale application;	Sun et al. [41]; Shaohua Li et al. [42]; Wang et al. [43]; Machado et al. [44]; Petit et al. [45]

## 4. Resveratrol Derivatives: Synthesis and Their Biological Activities

From a chemical point of view, the phytoalexin resveratrol (RSV) 3,4,5-trihydroxy-*trans*-stilbene (**I**) is the aglycon of the glycoside transpolidatin, also known as piceid, and chemically named 3,4,5-trihydroxystilbene-3-*O*-β-mono-D-glucoside. It is frequently found in numerous foods, fruits, and beverages and is released in the intestine after oral administration. Several researchers have confirmed that both glucoside and aglycon have numerous therapeutic effects, such as antidiabetic, antioxidant, anticarcinogenic, α-glucosidase (α-GLU) inhibition, and neuroprotective effects [47,48].


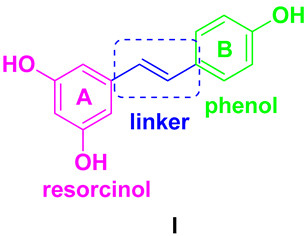


**Figure 3:**
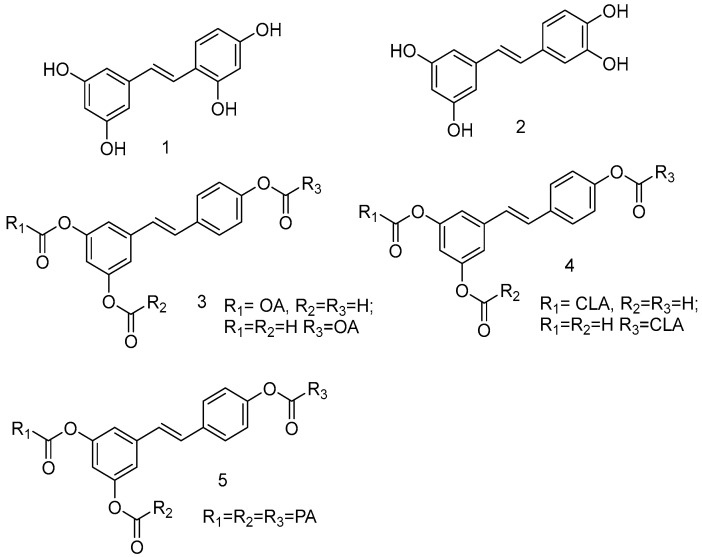
Hydroxylated resveratrol derivatives.

As a product of hydrolysis of polydatin, RSV is a smaller molecule, very close to a “hit” with a Log P of 3.06, a molecular weight of 228.25, and three hydrogen bond donor groups, tending to be well optimised. Due to its chemical structure (**I**), we recognized three main regions available for chemical modification and the synthesis of RSV derivatives, namely, the resorcinol (core A), the phenol (core B), and the ethene double bond (linker). Several modifications were addressed to add more OH groups, both on the A and B cores, with the scope to improve water solubility and/or pharmacological activity. In particular, the number and position of hydroxyls are crucial for antioxidant activity, as well as influencing pharmacokinetics. Oxyresveratrol (1) and piceatannol (2) (Figure 3), naturally occurring substances, showed better water solubility, faster absorption, and higher bioavailability. Piceatannol treatment in in vitro experimental models of AD reduced intracellular accumulation of ROS and apoptosis in the PC12 cell line, showing stronger protective effects against neuronal death than RSV [49]. On the other hand, the antiproliferative effects shown by RSV across three esters, namely, mono-RES-OA (3), mono-RES-CLA (4), and tri-RES-PA (5) (Figure 3), significantly reduced tumor cell viability up to 23% at concentrations of 25, 10, and 50 μg/mL, respectively, which turned out to be particularly interesting [50].

**Figure 4:**
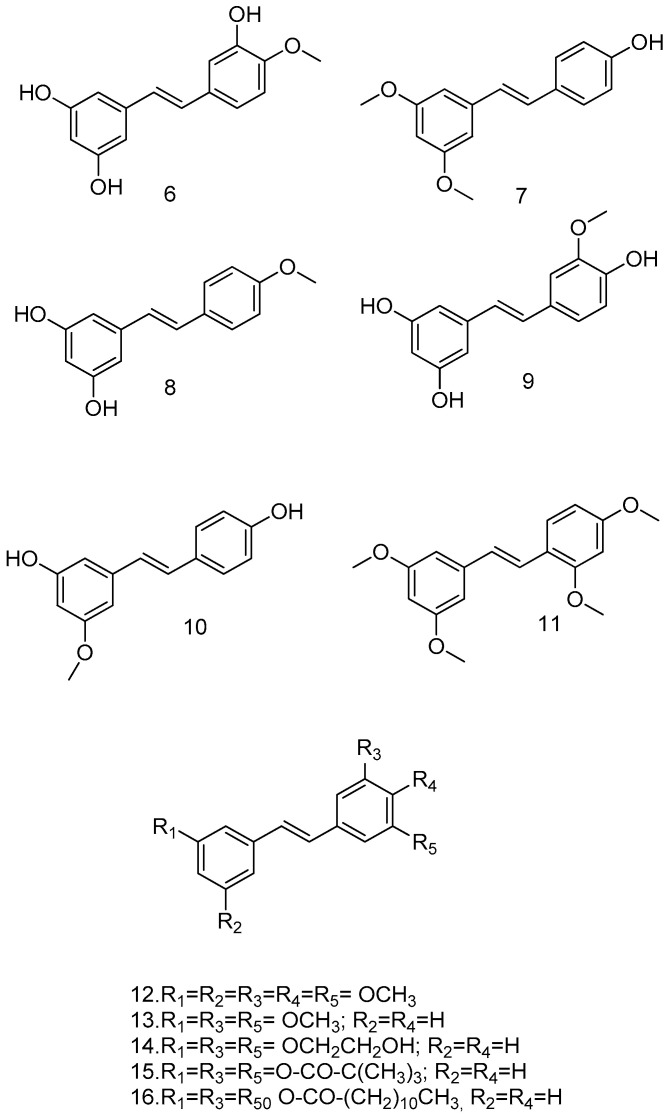
Methoxylated resveratrol analogs.

*O*-methylation of RSV can influence its metabolic stability and is the simplest and most commonly used organic and bio-organic reaction. Yoojin Chong et al. used a two-culture system for this purpose. The first cell synthesized resveratrol, while the second cell containing resveratrol modification genes synthesized the resveratrol derivatives, namely, isorhapontigenin (6), pterostilbene (7), 4-methoxyresveratrol (8), and rhapontigenin (9) (Figure 4). In this study, RSV and its methoxylated derivatives were evaluated for their anti-inflammatory activity. Pterostilbene showed the highest yield and the best anti-inflammatory activity. The methylation of hydroxyl groups at the 3 and 5 positions of the resorcinol moiety, was considered. This major structural change increases lipophilicity, improves oral absorption and cellular uptake, increases bioavailability, and reduces metabolism, and it is considered a less suitable substrate for human sulfotransferases than RSV. Pterostilbene may have a significant anti-inflammatory effect in vivo compared to RSV [51].

Pterostilbene has shown interesting neuroprotective effects in numerous in vitro assays and has been shown to improve cognitive function in animals in the radial arm water maze tested; the low toxicity of pterostilbene in animal models and humans is well documented. The best performance of pterostilbene can be attributed to its increased lipophilicity compared to RSV due to the simple replacement of the 3′- and 5′-hydroxyl groups with a methoxy group [52].

Experimental data suggest that methoxylated RSV analogs pinostilbene (10), pterostilbene (7), which also inhibits replication of the SARSCoV-2 virus in vitro, and tetra methoxystilbene (TMS) (11) (Figure 4) are more potent antitumor agents than the parent compound. In particular, TMS (11) was not only found to be a selective antiproliferative agent against the malignant MCF-7 cell line but also a chemopreventive agent in non-malignant breast cells [53].

**Figure 5:**
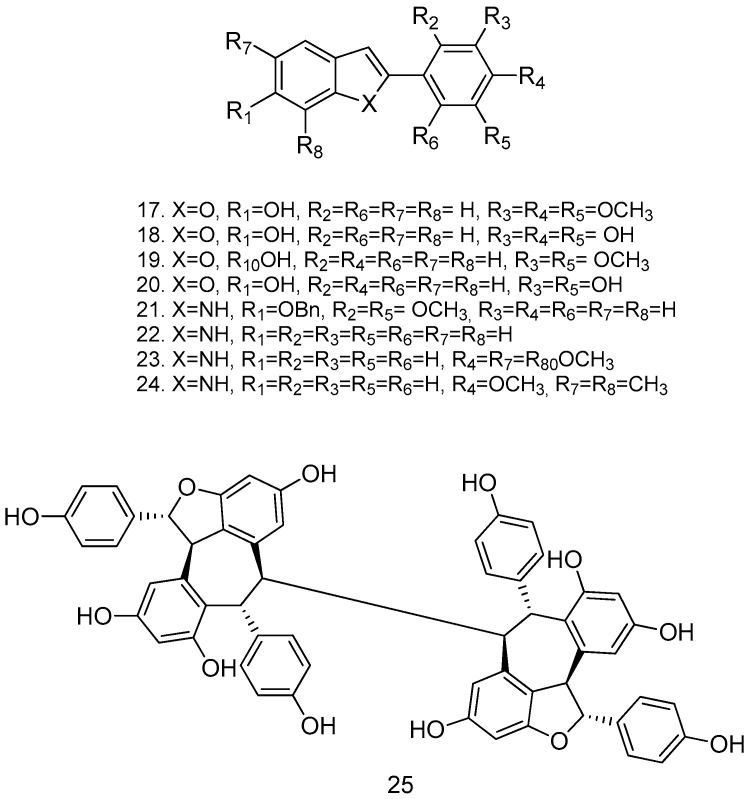
Cyclic derivatives of resveratrol.

Due to the presence of free hydroxyl groups, RSV rapidly undergoes conjugation with glucuronic acid and sulfation via PAPS (phosphoadenosylphosphosulphate); in fact, its half-life is very short (about 14 min when administered intravenously). To overcome this limitation, a series of methoxylated, acylated (Figure 4), and cyclic compounds were synthesized (Figure 5), and their antitumor activity was tested in human colon cancer tumor cells (HT-29) and in a pancreatic cancer cell line (MIA PaCa-2). Compounds **12**, **13**, **14**, and **15** showed significant growth reduction in both cell lines; compounds **17**, **18**, and **21** showed marked cytotoxic activity against HCT116 (KRas mutant) at 20 μM in vitro; and arylbenzofuran 17, which has a multi-target profile, could be considered a success in the design of new compounds with potential activity against colorectal cancer [54].

In another study, the antiplatelet and antiproliferative activity of several methoxylated RSVs was evaluated. The RSVs were obtained through the preparation of 3,5-dimethoxybenzylphosphonate, in which a benzoic acid is reduced with LiAlH_4_ to give benzyl alcohol, which is then reacted with CBr_4_ in the presence of PPh_3_, and the resulting bromide is then reacted with PO(OEt)_3_ to give the desired phosphonate. A Wittig reaction of the phosphonate with 4-methoxybenzaldehyde gives trimethoxyresveratrol. The most potent derivative observed was the 4-methoxy derivative, which exhibited approximately 2.5 orders of magnitude greater antiplatelet activity against TRAP-induced platelet aggregation, suggesting its potential as an antiplatelet agent. Using in silico docking simulations, all compounds were found to have a binding mode comparable to vorapaxar [55].

The cyclic derivatives of RSV also occur naturally. A bioassay-guided fractionation campaign on the leaf extracts of two Papua New Guinea rainforest plants, *Anisoptera thurifera* and *A. polyandra*, identified (-)-hopeaphenol (25), together with other less active stilbenoids, as putative inhibitors of type-III secretion in the Gram-negative pathogens *Yersinia pseudotuberculosis* and *Pseudomonas aeruginosa* Caroline E [56]. Preclinical studies have shown the promising therapeutic potential of RSV prodrugs such as 3,5,4-tri-*O*-acetylresveratrol (TARES) and the piceid acylated prodrug [resveratrol-3-*O*-(60-*O*-octanoyl)-b-D-glucopyranoside] in a model of Huntington’s disease (HD) [57].

**Figure 6:**
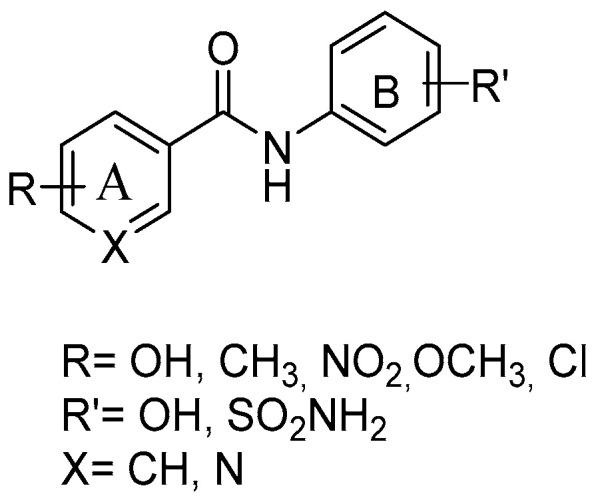
General structure of resveratrol amide derivatives.

With the aim of synthesizing elective COX-2 inhibitors as a reliable alternative to tNSAIDs, several amide RSV derivatives were prepared (Figure 6), starting from a general structure, including the amide moiety as substituted by the ethylenic linker, and adding a sulfonamide group at nucleus B—key features of potent coxib compounds. Condensation *via* acyl chloride activated reagent or acetone, and HATU and Et_3_N, afforded the final amides. The compound with an *o*-diphenol hydroxyl group in ring A showed a mild selective and a highly potent inhibitory effect on the COX-2 enzyme (COX-2 IC_50_ = 0.42 μM; SI = 83) without any gastric ulceration. A molecular docking study showed that the o-diphenol hydroxyphenyl moiety could penetrate deep into the COX-2 side pocket and form hydrogen bonds with Gln 192, Ile 517, and Phe 518 [58].

**Figure 7:**
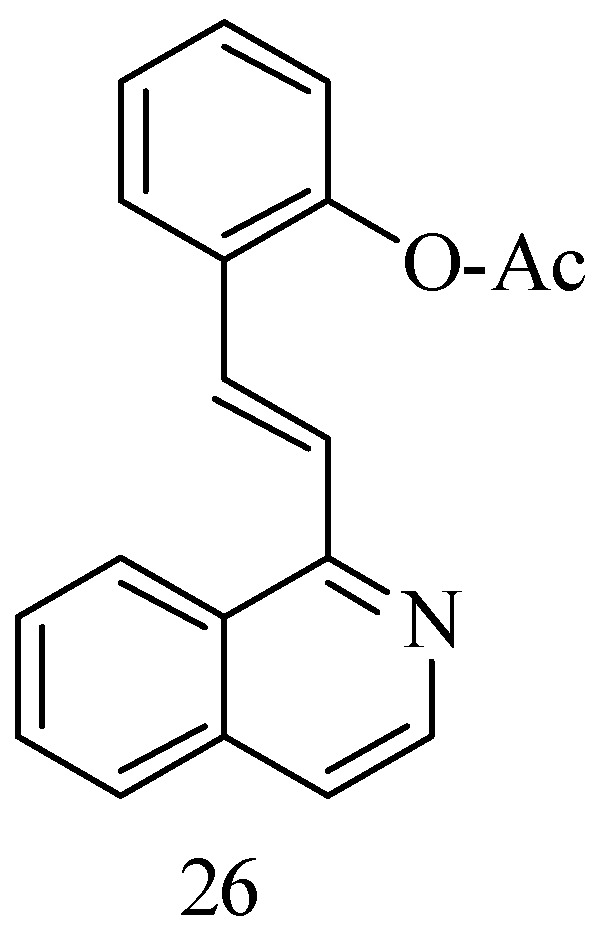
1-styrenyl isoquinoline derivatives.

Bearing in mind that the polyhydroxy and arylethylene motifs of RSV are key to its bioactivity, a series of new anticancer RSV 1-styrene–isoquinoline derivatives have been synthesized to maintain proper water solubility by replacing the resorcinol ring with an isoquinoline motif, which has the ability to enhance water solubility due to the presence of a nitrogen atom. Isoquinoline is also found in a large number of natural products with anticancer properties. The compounds were obtained via intermolecular nucleophilic addition of isoquinoline and a suitably substituted aldehyde (Figure 7). The best compound obtained, the 1-OAc substitute **26**, showed significant antiproliferative activity to Hun7 and SK-Hep-1 cells (2.52 μM and 4.20 μM, respectively) [59].

**Figure 8:**
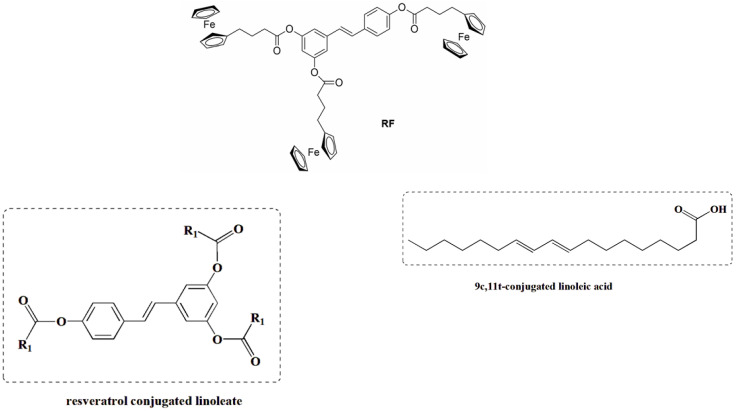
Ferrocene-containing triacyl derivative of resveratrol, RF, and resveratrol-conjugated linoleate.

Esterification modification is an effective method to improve the lipid solubility of RSV; to this end, a RSV conjugated linoleate (Figure 8) was synthesized for the first time from RSV and 9c,11t-conjugated linoleic acid (9c,11t-CLA) using *N*,*N*’-carbonyldiimidazole (CDI) as a catalyst. The final compound was identified as a triester structure with high conversion (96.9%) and yield (65.3%) using the following reaction conditions: 9c,11t-CLA molar ratio of 1:3; 9c,11t-CLA/CDI molar ratio of 1:1; modification time of 2 h; and 9c,11t-CLA activation time of 30 min. Compared to RSV, the triester has significantly improved lipid solubility, lower acid value, and higher thermal stability [60].

**Figure 9:**
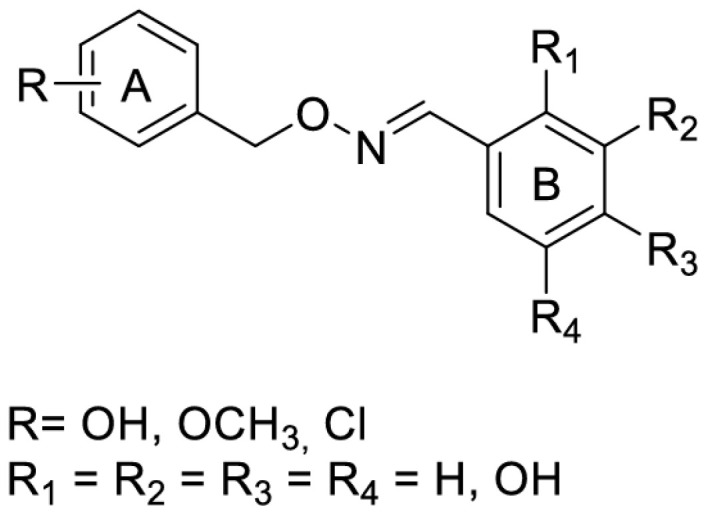
Resveratrol-like compounds.

Another strategy to overcome the poor bioavailability of RSV was to synthesize RSV derivatives containing a ferrocene moiety instead of a benzene ring, as well as a novel ferrocene–resveratrol conjugate with a trimethylene chain between the ferrocene and RSV moieties (Figure 8). In general, RSV derivatives and analogs were more effective compared to RSV in terms of several pharmacological and pharmacokinetic parameters. In this case, the RF compound is non-toxic to non-cancerous ovarian cells and has antioxidant properties, which is a significant improvement over the parent compound [61]. RSV is a known activator of sirtuin enzymes (SIRTs). SIRT1, in particular, is involved in cardiovascular homeostasis through the transcriptional inhibition of pro-inflammatory genes via the modulation of the nuclear factor kappa B (NF-κB) pathway. Using a computational protocol to identify the most promising derivatives from an in-house chemical library, several orally bioavailable RSV derivatives have been discovered. The chemical modifications of the in-house library included methoxylation and chlorination at different positions of both cores A and B and the new O-N bond (Figure 9). All modifications showed an impressive improvement in lipophilicity and better bioavailability with respect to RSV [62].

**Figure 10:**
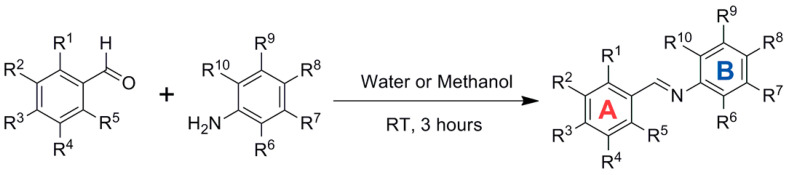
Synthesis of imine resveratrol analogs.

Activation of Nrf2 by RSV is thought to protect against phase I enzyme-activated carcinogens and associated carcinogenicity via the transactivation of antioxidant and phase-II detoxifying enzymes. New imine RSV analogs that mimic this effect with improved bioavailability and higher potency in activating Nrf2 have been synthesized by replacing the C=C bond of the resveratrol linker moiety with a C=N bond (Figure 10) and adding more hydroxy, methoxy, and halogen groups.

The hydroxyl substitution on ring B, i.e., R6=OH and R8=OH, could improve the Nrf2 induction. The meta-OH substitution on ring A improved the activity, and the ortho-OH substitution on ring B led to a three-fold increase in luciferase activity. In contrast, methyl substitution on ring B in either position had no significant—or even negative—effect on ARE luciferase activity. The preliminary SAR concluded that the 6-OH substituent group plays the crucial role in Nrf2 activation, while substitutions on ring A contribute as auxiliary cofactors [63].

**Figure 11:**
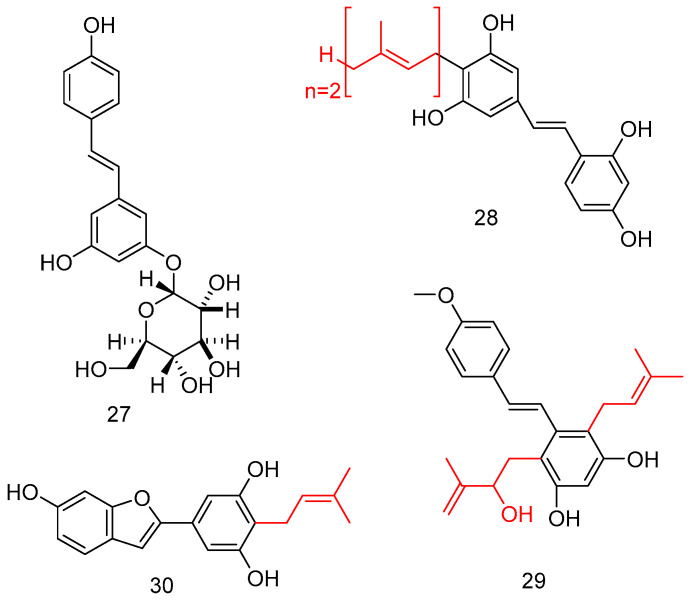
*O*-Glucoside and prenylated (red color) stilbene analogs.

Considering the poor bioavailability of natural stilbenes, including RSV, as well as UV instability, the introduction of a sugar moiety on a stilbene backbone improved the water solubility, as well as lowering the toxicity and improving the biological profile. The total synthesis of resveratrol 11-*O*-*β*-glucoside **27** was attempted via selective glycosylation of 3,5-dihydroxybenzaldehyd with a glycosyl donor, and under Wittig conditions, the key double bond of stilbene was constructed (Figure 11) [64].

Interestingly, prenylated stilbenoids, despite several being naturally occurring, were difficult to extract; in this case, has been performed several enzymatic reactions to obtain compounds like **28**–**30**, endowing potent α-glucosidase inhibitory activity (Figure 11) [65].

**Figure 12:**
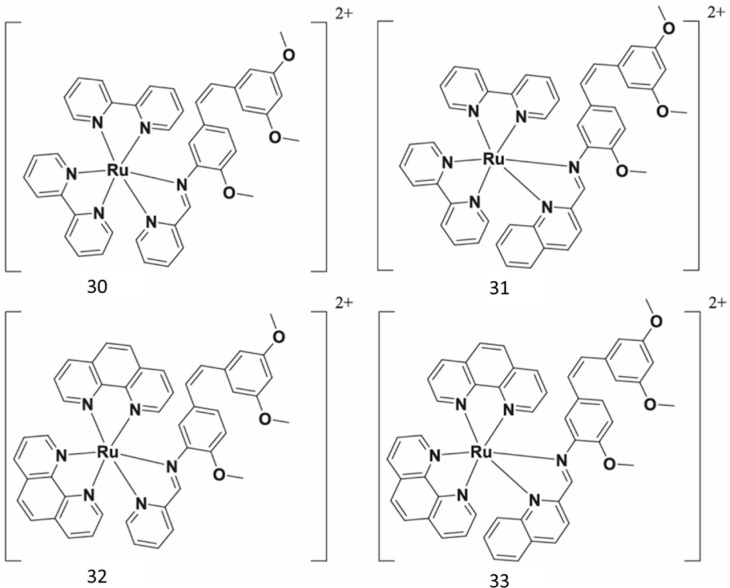
Resveratrol rutenium complexes.

Ru(II) complexes have been synthesized as novel photosensitizers, beginning with aminoresveratrol as ligand (Figure 12). A mechanism study revealed that **33** could inhibit cancer cell migration, invasion, and cancer stemness without significant toxicity [66].

Experimental studies revealed that RSV monoesters, synthesized through esterification with lipophilic groups, improved the lipophilicity, antioxidant activity, and bioavailability of the parent compound. In Dami Li et al. [67], seven 3-resveratrol monoesters (3-RC2:0–18:0) and seven 4′-RC2:0–18:0 were prepared, and antioxidant capacity and bioaccessibility were innovatively studied, focusing on substitution and acyl chain length effects. 3-RC2:0–18:0 was found to have stronger antioxidant effects than 4′-RC2:0–18:0, but 4′-RC2:0–8:0 had better oxygen radical absorption capacity than 3-RC18:0. 3-RC2:0 even showed better ABTS radical scavenging capacity than Trolox. However, 3-RC2:0/RC4:0 displayed higher antioxidant efficacy. 3-RC2:0/16:0 exhibited exceptional antioxidant capacity and digestive stability. These findings indicate resveratrol derivatives’ potential in lipid-based foods, pharmaceuticals, and cosmetics.

**Figure 13:**
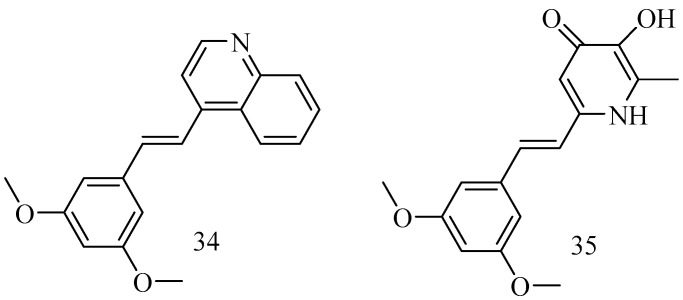
Heterocycles derivatives of RSV.

Ischemic stroke is a neurological disorder that often results in significant disability or mortality. RSV has been recognized for its potent neuroprotective properties, but its clinical application is limited by low oral bioavailability and instability. (*E*)-4-(3,5-dimethoxystyryl) quinoline (**34**) is an RSV derivative featuring a structural modification that substitutes RSV’s 3,5-dihydroxyl groups with 3,5-dimethoxy groups and its phenolic group with a quinolinyl moiety. Compound **34** was shown to inhibit neuronal apoptosis, attenuate oxidative stress, and enhance mitochondrial function [68]. The deferiprone–resveratrol hybrid (**35**) (Figure 13) 2-(3,5-dihy droxystyryl)-5-hydroxy-1-ethylpyridin-4(1 *H*)-one is chemically synthesized by combining deferiprone (DFP) and resveratrol (RsV) and shows an iron-chelating property along with antioxidant activity, which useful to reduce the high amount of iron in β-thalassemia patients that leads to oxidative stress and organ dysfunction [69].

**Figure 14:**
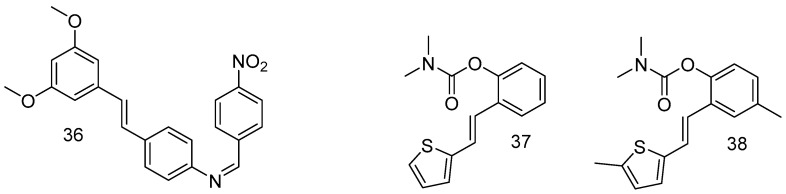
Resveratrol-derivative surrogates.

In order to improve the bioavailability of RSV through the modification of hydroxy groups, among all synthesized compounds, a substantial chemical modification afforded surrogates of RSV, such as the resulting compound **36** (Figure 14), which is a promising therapeutic agent against Alzheimer’s disease [70].

Other groups of resveratrol-based carbamates were synthesized, and compounds **37** and **38** were found to be the most active, representing potential selective BChE inhibitors as new therapeutics for neurological disorders [71].

**Figure 15:**
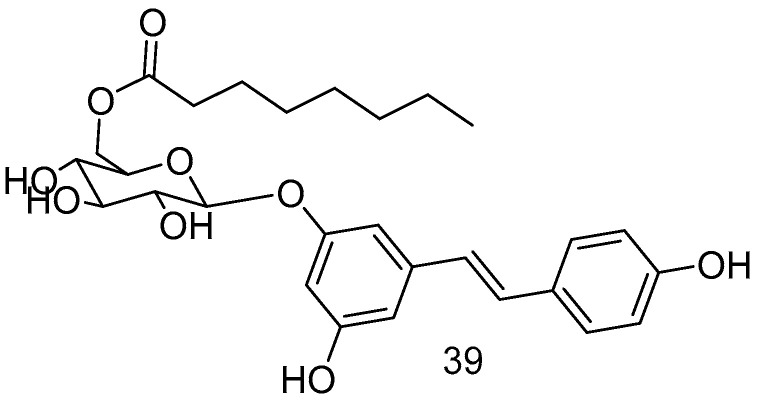
Picetannol derivative.

The piceid octanoate **39** (Figure 15), obtained through the esterification of the -CH_2_OH moiety of glucose in the glycoside derivative of RSV, named picetannol, showed remarkable delayed photoreceptor degeneration in the retinitis pigmentosa model, demonstrating superior efficacy to RES [72].

**Figure 16:**
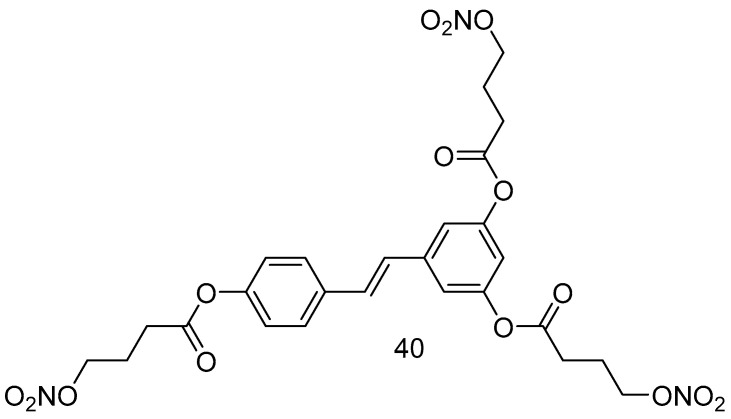
Resveratrol nitroderivative.

Giuseppe Guglielmini et al., in 2024, aimed to synthesize and characterize a new nitro derivative of resveratrol, trinitroresveratrol (**40**) (Figure 16), for its potential nitric oxide-donating and antiplatelet effects [73]. The synthesis of the reported compound was performed under condensation conditions starting from 4-bromo-butanoic acid in dry DCM at 0 °C, DCC/DMAPP, and RSV.

Finally, a new study reported the creation of resveratrol–piperazine cocrystals using multi-sound (US) and microwave-assisted (MW) methods. These methods have been used together with solution and slurry-based approaches to study how the synthesis process affects the properties of the cocrystal and whether the processes can be intensified. The potential of these cocrystals is represented by the unique properties of their components, resveratrol and piperazine, which could also be used in veterinary medicine. Resveratrol has been shown to fight off microbes, viruses, and cancer cells. Piperazine has been found to treat parasitic infections [74].

## 5. Advanced Drug Delivery Strategies for Resveratrol

Resveratrol (RSV) has significant therapeutic potential; however, its limited aqueous solubility and rapid metabolism severely hinder its bioavailability. In this regard, controlled drug delivery systems could offer an effective solution by which to overcome these limitations, allowing for a prolonged release of the drug, maintaining therapeutic concentration over time, facilitating precise targeting, and optimizing bioavailability. Among the various systems, solid lipid nanoparticles (SLNs)—polymeric, mesoporous, metallic, and dentrimeric—function as effective delivery systems able to improve the stability, bioavailability, and pharmacokinetic properties of resveratrol, and are therefore suitable for the its administration [75,76]. Cruz et al. developed and characterized resveratrol-loaded solid lipid nanoparticles (RSV-SLN) to improve its skin distribution. They formulated SLNs using stearic acid, soy phosphatidylcholine, polysorbate 80, cetyltrimethylammonium bromide, and poloxamer 407, varying heating temperatures and homogenization techniques. Stability was evaluated for 90 days by monitoring the organoleptic properties, hydrodynamic diameter, polydispersity index, and zeta potential. Encapsulation efficiency, which reached approximately 70%, and skin permeability studies confirmed the ability of SLNs to facilitate RSV release through the skin. These results suggest that SLNs are promising carriers for RSV skin delivery, offering increased stability and sustained release, thus representing a valuable strategy for topical applications aimed at exploiting the therapeutic benefits of RES for the skin [77].

To improve the pharmacokinetic profile of RSV, Das and colleagues developed RSV-loaded nanostructured lipid carriers (NLCs) (RSV-NLCs) and evaluated their toxicity against human ovarian cancer cells in a rat model. Synthesized via solvent injection, the NLCs showed a size of 177 nm, a polydispersity index of 0.25, and an entrapment efficiency of more than 80%. These carriers also demonstrated stability for three months at 4 °C and 40 °C. In vitro cytotoxicity assays (MTT method) and cellular uptake studies on human ovarian cancer cell lines revealed time-dependent cytotoxic effects and significantly higher cellular accumulation of RSV compared to free-drug administration. Pharmacokinetic analysis in rats following intravenous administration of RSV-NLC showed sustained plasma levels of RSV for at least 48 h, in contrast to the rapid clearance of the RSV solution. Furthermore, tissue distribution studies in rats indicated a significant increase in RSV accumulation in the liver, lung, and ovaries within 48 h of RSV-NLC administration, highlighting their potential for targeted therapy in ovarian cancer [78]. Qu et al., designed and fabricated functionalized nanoparticle drug delivery systems based on polyaminoamine dendrimers (PAMAMs) for the targeted delivery of resveratrol in the treatment of hepatocellular carcinoma [79]. The fifth-generation PAMAM dendrimers (G5) were modified with galactose (G5-Gal) via coupling reactions, followed by the physical encapsulation of resveratrol to create glycosylated dendrimers (G5(RSV)-Gal). The resulting G5-Gal and G5(RSV)-Gal complexes were characterized using 1H NMR spectroscopy, zeta potential, size analysis, and UV spectrophotometry. Hepa1–6 murine hepatoma cells served as a model by which to evaluate the targeting efficacy of G5-Gal to hepatoma cells. Subsequently, the CCK-8 assay evaluated the impact of G5(RSV)-Gal on normal hepatocytes and its cytotoxicity against various hepatoma cell lines. The combined results suggest the potential for the development of targeted nanomedicines and therapeutic strategies for hepatocellular carcinoma.

Cyclodextrins (CDs) have also emerged as attractive carriers due to their ability to improve the stability, solubility, and bioavailability of various active ingredients [80,81,82,83,84]. These cyclic oligosaccharides, characterized by a hydrophilic exterior and a hydrophobic cavity, can form inclusion complexes with poorly soluble drugs, thus improving their pharmacokinetic profile and therapeutic efficacy.

Yu et al. used molecular simulations to evaluate the release of cyclodextrin-resveratrol inclusion complexes on the surface of the lipid bilayer of cells [85]. Simulations indicated that the structural orientation of resveratrol within the cyclodextrin significantly influences the release kinetics. Resveratrol can form inclusion complexes with β-CDs in two orientations: M-form (mono-hydroxyl group towards the primary edge) and D-form (di-hydroxyl group towards the secondary edge). The results demonstrate that the M-form complex facilitates the release of RSV more efficiently than the D-form. Furthermore, the β-CD/RSV complex showed higher stability and release efficiency at the lipid membrane than γ-CD/RES and α-CD/RSV, suggesting that the cavity size of β-CD is optimal for the release of resveratrol.

In another study, resveratrol was incorporated into hydroxypropyl-β-cyclodextrin complexes and subsequently formulated into a Pluronic F-127 hydrogel for wound-healing therapy [86]. The wound-healing potential of these complexes was assessed using a fibroblast scratch assay, which showed a trend toward the increased efficacy of resveratrol after complexation. The antimicrobial activity of resveratrol, both in aqueous dispersion and within the complexes, was evaluated against methicillin-resistant Staphylococcus aureus (MRSA), Escherichia coli, and Candida albicans. The results revealed a two-fold reduction in the minimum inhibitory concentration (MIC) and a greater inhibition of MRSA metabolic activity following treatment with resveratrol in the complexes compared to the drug in suspension. Furthermore, the incorporation of these complexes into the Pluronic hydrogel ensured efficient drug release and adequate viscoelastic properties. The formulated hydrogel demonstrated excellent biocompatibility, as confirmed by skin irritation tests in rabbits. These results suggest that the Pluronic hydrogel containing resveratrol within hydroxypropyl-β-cyclodextrin complexes represents a promising topical formulation for further wound therapy studies.

**Figure 17:**
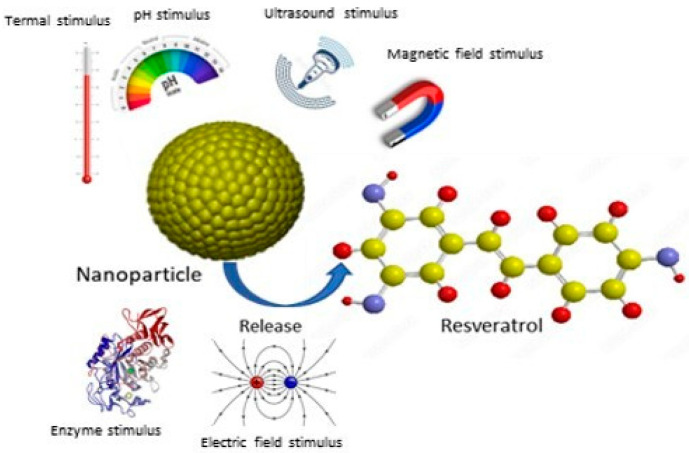
Resveratrol release from stimuli-responsive nanoparticles.

The encapsulation of stimuli-responsive nanoparticles also represents a particularly promising strategy. This approach improves the solubility, absorption, and biocompatibility of resveratrol, maximizing its therapeutic efficacy. Ultimately, the development of such advanced pharmaceutical formulations is essential to unleash the full clinical potential of this valuable compound (Figure 17).

In particular, the encapsulation of stimuli-responsive nanoparticles represents a particularly promising strategy by which to fully exploit the clinical potential of this valuable compound (Figure 11).

Indeed, such nanoparticles show significant selective uptake by target cells, improving their permeability and retention within target tissues and cellular uptake with a reduction in systemic toxicity [87,88,89,90,91,92]. These carriers can be designed to respond to specific internal or external triggers, including pH, temperature, light, magnetic fields, and ultrasound [93]. pH-responsive nanoparticles are particularly valuable in cancer therapy as RSV release can be triggered by the acidic pH characteristic of the tumor microenvironment, thus minimizing release into healthy tissues. For example, in 2019, Zheng and colleagues developed a ferritin-based pH-induced reversible assembly system (PIRAS) to enhance RSV release at tumor sites [94]. The ferritin sphere surface modified with RGD peptide demonstrated stability in neutral and alkaline environments (pH > 7.4) and selectively released RSV under acidic conditions (pH < 7.4). Furthermore, the RGD-mediated targeting effect facilitated high uptake of RV@Ft-RGD by A549 tumor cells, leading to lysosomal accumulation and the subsequent release of RSV into the cytoplasm. This resulted in significant tumor cell killing and a significant apoptosis-promoting effect compared to free RSV. In vivo studies confirmed that RV@Ft-RGD showed remarkable tumor suppression without significant systemic toxicity. The enzyme-activated release of RSV is another promising approach. This can be achieved by incorporating enzyme-cleavable linkers within the nanoparticle matrix. For example, metalloproteinases, which are often overexpressed in aggressive and metastatic tumors, can cleave specific bonds in nanoparticles, leading to localized release of RSV at the tumor site [95].

With regard to thermoresponsive nanoparticles, De Luca et al. aimed to improve the efficacy of eye drop solutions for dry eye syndrome (DED) by designing an in situ thermogelling formulation containing RSV-loaded polymeric nanoparticles. RSV-loaded cationic acetylated polyethylene glycol nanoparticles were prepared by nanoprecipitation and incorporated into a thermoresponsive hydrogel (RSV@Tgel) based on Poloxamer 407 [69]. The rationale was that the thermoresponsive polymer would prolong the drug-cornea contact time, minimizing drug loss due to ocular drainage. The results indicated that RSV@Tgel could serve as an excellent adjuvant in the treatment of DED due to its protective effects against inflammation and oxidative stress, as well as the reduced frequency of eye drops administration [96].

Fang and colleagues developed a pegylated cyclodextrin (CD)-based nanoplatform (PCP) for the localized delivery of RSV nanomicelles (RSV-NM) for the treatment of inflammatory osteolysis [97]. Recognizing that excessive osteoclast activity and reactive oxygen species (ROS) play a crucial role in inflammatory bone loss, and that RSV can inhibit this process by scavenging ROS, they designed a ROS-responsive delivery system. By incorporating the phenylboronic acid ester into the nanoplatform, the resulting RSV-NM showed excellent biocompatibility and significantly improved the solubility and stability of RSV compared with free RSV. In vitro studies demonstrated the efficacy of RSV-NM in suppressing osteoclast formation, suggesting its potential as a therapeutic agent for inflammatory osteolysis [97].

Another interesting application of resveratrol (RES) concerns the treatment of Alzheimer’s disease (AD). In this regard, Abbas and colleagues employed chitosan-coated bilosomes loaded with RSV and superparamagnetic iron oxide nanoparticles (SPIONs) to reach the brain through the intranasal olfactory mucosa following the application of an external magnetic field [98]. These bilosomes were then embedded in sodium alginate/PVP wafers.

Both the bilosomes and the wafer formulation were characterized in vitro for size, porosity, morphology, RSV entrapment, and release efficiency.

In vivo studies were performed on AD mice, comparing the effects of SPION, conventional bilosomes, and RSV suspension in relation to cognitive and mnemonic functions.

The results obtained demonstrated the superior efficacy of SPION bilosomes compared to other formulations. This finding could be attributed to the enhanced therapeutic effects of RSV achieved by its nanoencapsulation and nasal administration, as well as the application of an external magnetic field that allowed for the targeted delivery of RSV to the brain.

Liping and colleagues synthesized a novel glutathione-responsive polymer (PRES) with potent antitumor properties [99]. This polymer, created through the condensation polymerization of resveratrol (RES) and 3,3′-dithiodipropionic acid, exhibits unique characteristics. PRES not only inhibits the growth of tumor cells but also spontaneously self-assembles into nanoparticles with an average diameter of approximately 93 nm. This nanoformulation was not only stable in blood circulation but also degraded in the high-glutathione environment of tumor cells. The results obtained suggest that the synthesized PRES platform holds great promise as a novel therapeutic strategy for cancer treatment.

In conclusion, the pivotal role of nanoparticles in the controlled release of resveratrol warrants significant attention. This innovative approach not only enables the triggered release of this valuable bioactive compound but also substantially enhances its bioavailability, effectively addressing the inherent limitations of direct resveratrol administration such as poor solubility and rapid degradation. Moreover, the capacity to induce resveratrol release in response to specific stimuli presents an exciting avenue for targeted drug delivery. This precise control holds the potential to revolutionize therapies for a spectrum of diseases, including cancer and cardiovascular disorders. Crucially, preclinical evidence robustly suggests that nanoparticle-based resveratrol delivery systems exhibit a commendable safety profile and biocompatibility in vitro, demonstrating a lack of significant toxicity in cellular models [100,101]. Indeed, animal studies further underscore the enhanced safety afforded by these systems compared to free resveratrol, indicating a reduced risk of adverse effects [102,103]. While clinical safety data for these novel delivery systems remain somewhat limited as human studies have predominantly focused on resveratrol itself and its known bioavailability challenges, the preclinical findings are highly encouraging regarding the inherent safety of the nanoparticle carriers. Although resveratrol itself is considered safe at moderate doses, continued clinical investigation into the long-term safety and potential interactions of these enhanced delivery systems is essential. Future safety evaluations should encompass comprehensive preclinical toxicology assessments (both in vitro and in vivo), thorough immunogenicity profiling, and formulation-specific testing to ensure a seamless and safe clinical translation. These compelling findings carry profound implications for the advancement of innovative resveratrol delivery strategies. By facilitating personalized therapeutic interventions, we can unlock the full therapeutic potential of this valuable bioactive compound and ultimately improve patient outcomes. The future of resveratrol (RSV) extraction, modification, and delivery lies in the continued development and optimization of green extraction methods, particularly those utilizing deep eutectic solvents (DESs). Looking ahead, DESs hold significant potential for the synthesis of novel RSV analogs with improved bioactivity and stability. Additionally, DESs can be utilized in advanced drug delivery systems to enhance RSV bioavailability and efficacy. These advancements not only support the shift towards greener extraction processes but also pave the way for more effective therapeutic applications of RSV in the pharmaceutical and nutraceutical industries. The integration of stimuli-responsive nanoparticles and other innovative delivery systems will further enhance the targeted delivery and controlled release of RSV, maximizing its therapeutic potential and minimizing adverse effects. Collectively, these strategies will contribute to the development of more effective and sustainable resveratrol-based therapies, ultimately improving patient outcomes and supporting the broader goals of environmental sustainability and public health.
